# Deletion of a Gene Encoding a Putative Peptidoglycan-Associated Lipoprotein Prevents Degradation of the Crystalline Region of Cellulose in *Cytophaga hutchinsonii*

**DOI:** 10.3389/fmicb.2018.00632

**Published:** 2018-04-03

**Authors:** Xifeng Wang, Zhiquan Wang, Xinfeng Bai, Yue Zhao, Weican Zhang, Xuemei Lu

**Affiliations:** ^1^State Key Laboratory of Microbial Technology, Shandong University, Jinan, China; ^2^Key Laboratory for Biosensors of Shandong Province, Biology Institute of Shandong Academy of Sciences, Jinan, China

**Keywords:** *Cytophaga hutchinsonii*, peptidoglycan-associated lipoprotein, cellulose degradation, crystalline region, integrity of the outer membrane

## Abstract

*Cytophaga hutchinsonii* is a gliding Gram-negative bacterium in the phylum *Bacteroidetes* with the capability to digest crystalline cellulose rapidly, but the mechanism is unclear. In this study, deletion of *chu_0125*, encoding a homolog of the peptidoglycan-associated lipoprotein (Pal), was determined to prevent degradation of the crystalline region of cellulose. We found that the *chu_0125* deletion mutant grew normally in regenerated amorphous cellulose medium but displayed defective growth in crystalline cellulose medium and increased the degree of crystallinity of Avicel. The endoglucanase and β-glucosidase activities on the cell surface were reduced by 60 and 30% without *chu_0125*, respectively. Moreover, compared with the wild type, the *chu_0125* deletion mutant was found to be more sensitive to some harmful compounds and to release sixfold more outer membrane vesicles (OMVs) whose protein varieties were dramatically increased. These results indicated that CHU_0125 played a critical role in maintaining the integrity of the outer membrane. Further study showed that the amounts of some outer membrane proteins were remarkably decreased in the *chu_0125* deletion mutant. Western blotting revealed that CHU_3220, the only reported outer membrane protein that was necessary and specialized for degradation of the crystalline region of cellulose, was largely leaked from the outer membrane and packaged into OMVs. We concluded that the deletion of *chu_0125* affected the integrity of outer membrane and thus influenced the localization of some outer membrane proteins including CHU_3220. This might be the reason why deletion of *chu_0125* prevented degradation of the crystalline region of cellulose.

## Introduction

Cell envelopes of Gram-negative bacteria consist of an outer membrane, an inner membrane, and a thin rigid layer of peptidoglycan located in the periplasm (Malanovic and Lohner, 2016). In order to maintain the integrity and stability of cell envelope, peptidoglycan interacts with many outer membrane and inner membrane proteins, one of which is the peptidoglycan-associated lipoprotein (Pal) (Parsons et al., 2006; Godlewska et al., 2009). Pal, widely distributed in Gram-negative bacteria, interacts with peptidoglycan through the C-terminal region and links to the outer membrane through the N-terminal lipid (Lazzaroni and Portalier, 1992). Pal is also an important component of the Tol-Pal complex consisting of an inner membrane sub-complex TolA-TolQ-TolR and an outer membrane associated sub-complex TolB-Pal (Lloubes et al., 2001). The Tol-Pal complex is considered to be involved in maintaining the proper structure and function of the cell envelope (Lloubes et al., 2001). Mutation in *pal* causes a defect in the integrity of the outer membrane, resulting in hypersensitivity to harmful compounds, leakage of outer membrane and periplasmic proteins, and formation of abundant outer membrane vesicles (OMVs) (Bernadac et al., 1998; Kowata et al., 2016). OMVs are released from the outer membrane into the extracellular milieu, carrying outer membrane proteins, periplasmic proteins, lipopolysaccharides, and phospholipids (Schwechheimer and Kuehn, 2015).

Cellulose, which is composed of the crystalline region and the amorphous region, is the most abundant biological resource on earth (Bayer and Lamed, 1992). To degrade cellulose, some aerobic bacteria secret free cellulases to the extracellular milieu and some anaerobic bacteria produce cellulosomes (Lynd et al., 2002; Bayer et al., 2004). *Cytophaga hutchinsonii* is a widely distributed aerobic Gram-negative bacterium from the phylum *Bacteroidetes*, which exhibits gliding motility over surfaces and digests cellulose rapidly (Stanier, 1942; Xie et al., 2007). The strategy for *C. hutchinsonii* to digest cellulose is different from the free-cellulase and the cellulosome mechanisms (Zhu and McBride, 2017). *C. hutchinsonii* appears to use a contact-dependent digestion strategy and most of the cellulase activities are cell surface associated, indicating the importance of outer membrane in degrading cellulose (Wilson, 2009; Ji et al., 2013). However, the mechanism of cellulose degradation by *C. hutchinsonii* is still poorly understood. Several outer membrane proteins, including CHU_1276, CHU_1277, and CHU_0170, were demonstrated to be indispensable to cellulose utilization (Ji et al., 2014; Zhu and McBride, 2014; Zhou et al., 2016). Recently, Wang et al. (2017) reported that the outer membrane protein CHU_3220 was necessary for the degradation of the crystalline region but not for the amorphous region of cellulose. These studies further indicated the importance of outer membrane proteins in cellulose utilization. However, other factors that may be related to cellulose degradation need to be explored.

As an important component in maintaining the outer membrane integrity, peptidoglycan-associated lipoprotein might influence the location and function of some outer membrane proteins, so it might play a role in cellulose degradation by *C. hutchinsonii.* In this study, *chu_0125*, encoding a putative peptidoglycan-associated lipoprotein, was deleted. The outer membrane integrity of *C. hutchinsonii* was evaluated by examining its sensitivity to some harmful compounds, the quantification of OMVs and the OMV proteins. The cellulose utilization ability of the Δ*0125* mutant was studied and the results showed that deletion of *chu_0125* prevented degradation of the crystalline region of cellulose.

## Materials and Methods

### Bacterial Strains, Plasmids, and Growth Conditions

*Cytophaga hutchinsonii* ATCC 33406 was grown in PY6K medium (6 g/L peptone, 0.5 g/L yeast extract, 1 g/L KNO_3_, 4 g/L glucose, pH 7.3), modified from PY6 medium (6 g/L peptone, 0.5 g/L yeast extract, 4 g/L glucose, pH 7.3) (Xu et al., 2012). To analyze cellulase activity, *C. hutchinsonii* was cultured in Stainer medium (1 g/L KNO_3_, 1 g/L K_2_HPO_4_, 0.2 g/L MgSO_4_⋅7H_2_O, 0.02 g/L FeCl_3_⋅6H_2_O, 0.1 g/L CaCl_2_, pH 7.3) (Stanier, 1942) plus 2 g/L glucose. *Escherichia coli* strains were grown in LB medium (10 g/L tryptone, 5 g/L yeast extract, 10 g/L NaCl, pH 7.0) at 37°C with shaking at 170 rpm. Antibiotics were used at the following concentrations: ampicillin (Ap), 100 μg/mL; erythromycin (Em), 30 μg/mL; chloramphenicol (Cm), 15 μg/mL. The strains and plasmids used in this study are listed in **Table 1**. Primers used in this study are listed in Supplementary Table S1.

**Table 1:** Strains and plasmids used in this study.

Strain or plasmid	Description^a^	Reference or source
***E. coli* strains**		
DH5α	Strain for gene cloning	TaKaRa
***C. hutchinsonii* strains**		
ATCC 33406	Wild type	ATCC
Δ*0125* strain	*chu_0125* deleted	This study
C*0125* strain	Complementation of Δ*0125* mutant with pCH0125	This study
Δ*1075* strain	*chu_1075* deleted	This study
Δ*3437* strain	*chu_3437* deleted	This study
Δ*0522* strain	*chu_0522* deleted	This study
Δ*0135* strain	*chu_0135* deleted	This study
Δ*1429* strain	*chu_1429* deleted	This study
**Plasmids**		
pTSK	Gene-targeting template plasmid carrying *ermF* flanked by two FRT site; Ap^r^ (Em^r^)	Wang et al., 2014
pCHF	Plasmid carrying *flp*; Ap^r^ (Cm^r^)	Wang et al., 2014
pCH	Vector used for complementation; Ap^r^ (Cm^r^)	Ji et al., 2014
pCH0125	A 2.2- kbp fragment spanning *chu_0125* ligated into *Sac*I and *Sal*I sites of pCH; *oriC*; Ap^r^ (Cm^r^)	This study

^a^*Antibiotic resistance phenotypes: Ap^r^, ampicillin; Em^r^, erythromycin; Cm^r^, chloramphenicol. Phenotypes in parentheses are expressed in C. hutchinsonii, and phenotypes not in parentheses are expressed in E. coli.*

### Construction of the *chu_0125* Deletion Mutant

Deletion of *chu_0125* (yielding the Δ*0125* mutant) was performed as previously reported (Wang et al., 2014). Briefly, a 2.4-kbp fragment spanning the three flanking genes (*chu_0122*, *chu_0123*, and *chu_0124*) and the first 175 bp of *chu_0125* was amplified with primers 0125H1F and 0125H1R. The fragment referred as H1 was digested with *BamH*I and *Kpn*I and cloned into pTSK digested with the same enzymes. A 2.0-kbp fragment spanning the two flanking genes (*chu_0126* and *chu_0127*) and the last 198 bp of *chu_0125* was amplified with primers 0125H2F and 0125H2R. The fragment referred to as H2 was digested with *Sal*I and *Sac*I and ligated into corresponding sites of pTSK, which flanked the *ermF-*FRT cassette opposite H1. The gene-targeting cassette was amplified with primers H1F and H2R, purified with a Cycle Pure kit (Omega, GA, United States) and transformed into 100 μL of competent cells of *C. hutchinsonii* by electroporation. The transformants were verified by PCR with two sets of primers, 0125UF/0125DR and 0125UF/0125UR. Then the correct transformants were used as parent strain for transforming the helper plasmid pCHF by electroporation to get the unmarked mutant. After incubation for about 15 days, the transformants were cultured without antibiotics to lose the pCHF. Then the cells were verified by PCR with primers 0125UF and 0125DR and the PCR products were sequenced to verify the scar sequence.

### Complementation of the Δ*0125* Mutant

The complementation of the Δ*0125* mutant was performed as described previously (Wang et al., 2014). Briefly, a 2.2-kbp fragment spanning *chu_0125*, 187 bp upstream of the start codon and 96 bp downstream of the stop codon, was amplified with primers 0125CF and 0125CR. The fragment was digested and ligated into the pCH plasmid to generate pCH0125. Then the plasmid was transformed into 100 μL of competent cells of the Δ*0125* mutant by electroporation. C*0125* refers to the complemented strain of the Δ*0125* mutant with pCH0125.

### Filter Paper Degradation Assay

Filter paper degradation assays were carried out as previously described (Ji et al., 2012; Wang et al., 2014). Briefly, equivalent amounts of middle exponential phase cells from Stainer medium were spotted on Whatman NO. 1 filter paper overlaid on Stanier medium supplemented with 10 g/L agar. The plates were incubated at 30°C to observe the degradation.

### Growth Analysis With Different Carbon Sources

When glucose was used as the carbon source, the growth curves were monitored by Bioscreen C analyzer (Oy growth curves Ab Ltd, Finland) (Bai et al., 2017a). Briefly, *C. hutchinsonii* strains were grown in Stainer medium to middle exponential phase and inoculated into 200 μL of Stanier, PY6, and PY6K medium in a sample plate. The plate was incubated at 30°C with shaking at medium speed, and the growth curves were monitored by the optical density at 600 nm every 3 h. When Avicel PH101 and regenerated amorphous cellulose (RAC) were used as the carbon sources, all the strains were incubated in 500 mL flasks at 30°C with shaking at 160 rpm. To measure the growth rates, the samples were taken out at indicated time points and centrifuged at 13,500 × *g* for 10 min to get the pellets. Then the pellets were resuspended in 0.2 M NaOH, boiled for 10 min and centrifuged at 13,500 × *g* for 10 min to get the supernatant. Total cellular proteins of the supernatant were quantified as described by Bradford (1976). The weight of residual Avicel was measured as described by Zhu et al. (2010).

### Measurement of the Crystallinity of Cellulose by X-Ray Diffraction (XRD)

Strains were grown in Stainer medium to middle exponential phase. 30 mL of the cells were harvested and washed twice by fresh Stainer medium, then resuspended in 1 mL Stainer and transferred into 30 mL Stainer medium supplemented with 4 g/L Avicel PH101 and cultured at 30°C with shaking at 160 rpm. Samples were taken at set intervals (0.5, 24, and 48 h). All the samples were centrifuged, resuspended in 0.2 M NaOH, boiled for 10 min, and centrifuged at 13,500 × *g* for 10 min to remove the supernatant. The pellets were then washed twice with distilled water and dried at 60°C overnight. The XRD of samples was observed with a D8 ADVANCE System Diffractometer (Bruker, Germany). The XRD crystallinity index (*CI*_XRD_) was calculated as described by Wang et al.: *CI*_XRD_ (%) = (*I*_002_ -*I*_am_)/*I*_002_ × 100%. *I*_002_ is the height of the crystalline peak at 22° and *I*_am_ is the intensity of the peak at 18° (Wang et al., 2017).

### Cellulase Activity Assay

Cellulase activity assays were carried out as previously described (Bai et al., 2017a; Wang et al., 2017). Briefly, cells were grown in Stanier medium and harvested at different growth phase. For intact cell samples, cell pellets were resuspended in Na_2_HPO_4_-KH_2_PO_4_ buffer (100 mM, pH 6.8). For cell-extract samples, cell pellets were resuspended in Na_2_HPO_4_-KH_2_PO_4_ buffer with 2% (vol/vol) Triton X-100, and then the cells were incubated at 4°C for 5 h. The protein concentration was quantified as described by Bradford (Bradford, 1976). 1% (wt/vol) sodium carboxymethyl cellulose (CMC-Na) and 2 mM *p*-Nitrophenyl β-D-glucopyranoside (*p*NPG) were used as substrates to measure carboxymethyl cellulase (CMCase) activity and β-glucosidase activity, respectively. Cellulase activity of the intact cells was the cellulase activity of the cell surface. Intracellular cellulase activity was equal to cellulase activity of the cell extracts minus that of the intact cells. All the measurements were carried out in triplicate.

### Disk Diffusion Susceptibility Test

Disk diffusion susceptibility test was performed as described by Bai et al. (2017b). *C. hutchinsonii* was grown in PY6K medium to middle exponential phase and spread evenly over PY6K agar plates. A 6 mm paper disk was placed on the center of the plate and 3 μL of reagents was added, followed by incubation at 30°C for 3 days. The inhibition zone diameters were determined by the average of three plates. The reagents for the test were sodium dodecyl sulfate (10%), ampicillin (100 μg/mL), dithiothreitol (2 mM), H_2_O_2_ (2%), and crystal violet (1%).

### Observation of OMVs by Scanning Electron Microscopy (SEM)

The samples of OMVs were prepared as previously described with some modification (Nur et al., 2013). The strains were grown in PY6K medium in a 24-well culture plate with coverslips on the bottom. After incubation for 4 days, the coverslips were removed and washed twice with distilled water, and then fixed with 2.5% glutaraldehyde at 4°C overnight. All the samples were dehydrated through a graded series of ethylalcohol and dried by a critical point dryer (EM CPD300, Leica, Germany) for SEM (Quanta 250 FEG, FEI, United States).

### Quantification of OMVs

The quantification of OMVs was measured as previously described (Stewart, 1980; Wessel et al., 2013; Gujrati et al., 2014). Briefly, cells were grown in PY6K medium to middle exponential phase and harvested by centrifugation at 5,100 × *g* for 10 min at 4°C. Then the supernatants were filtered through a 0.45 μm-pore-size PVDF filter (Sangon Biotech, Shanghai, China) to remove all the cells. Cell-free supernatants were then centrifuged at 200,000 × *g* for 1 h to get vesicle pellets, followed by washing twice with PIPES (50 mM). Cell-free supernatants from which OMVs had been removed were labeled as OMV-free supernatants. For quantification, vesicle pellets were resuspended in MV buffer (50 mM Tris, 5 mM NaCl, 1 mM MgSO_4_⋅7H_2_O, pH 7.4) and extracted 1:1 with chloroform. The organic layers were removed into a new tube, combined with an equal volume of ammonium ferrothiocyanate solution (27.03 g/L FeCl_3_⋅6H_2_O, 30.4 g/L NH_4_SCN), and vortexed to guarantee the phospholipids of OMVs mixed completely with ammonium ferrothiocyanate solution. Then the organic layers were removed into a new tube, dried under N_2_ gas, resuspended in chloroform. The absorbance of ammonium ferrothiocyanate and phospholipids complex at 470 nm was analyzed.

### Preparation of Outer Membrane Proteins

Outer membrane proteins were prepared as described by Ji et al. (2014) and Wang et al. (2017). Briefly, the strains were grown in PY6K medium to middle exponential phase and harvested by centrifugation at 5,100 × *g* for 10 min at 4°C. Then the pellets were washed with PIPES (50 mM), resuspended in PIPES (50 mM) with 0.5 M NaCl, and incubated at 4°C for 20 min with shaking at 150 rpm. The cells were removed by centrifugation at 13,500 × *g* for 20 min at 4°C, and the supernatant containing the buffer-washed proteins was ultracentrifuged at 100,000 × *g* for 30 min at 4°C. Then the pellets were resuspended in PIPES (50 mM) as the outer membrane proteins, and the outer membrane proteins were subjected to SDS-PAGE. The weakened and missing protein bands in the profile of the Δ*0125* mutant compared with that of the wild type were cut and analyzed by matrix-assisted laser desorption/ionization time-of-flight (MALDI-TOF) mass spectrometry.

### Detection and Localization of CHU_3220

Outer membrane proteins were prepared as described above. OMVs were isolated as described above and resuspended in PIPES (50 mM) to obtain the OMV proteins. Extracellular proteins were prepared as described previously (Nandakumar et al., 2006). Briefly, cells were removed by centrifuging at 5,100 × *g* for 10 min. The supernatants were collected and filtered through a 0.22 μm-pore-size PVDF filter (Sangon Biotech, Shanghai, China). Cell-free supernatants and OMV-free supernatants were precipitated by treating with 10% (vol/vol) TCA (trichloroacetic acid) in ice for 30 min. Then the precipitates were collected by centrifugation at 13,500 × *g* for 10 min at 4°C and washed three times with pre-cooled acetone to remove traces of TCA. Finally, the precipitated proteins were solubilized in PIPES (50 mM). The localization of CHU_3220 was detected by Western blotting analysis using the CHU_3220 antibody as described by Wang et al. (2017).

### Bioinformatics

The genome sequence of *C. hutchinsonii* was obtained from the NCBI database^1^. CLUSTALW^2^ was used for the multiple alignment of related Pals. T-COFFEE^3^ and MUSCLE^4^ were applied to verify the CLUSTALW results. BoxShade^5^ was a tool to shade multiple alignment files.

## Results

### Bioinformatic Analysis of CHU_0125

**FIGURE 1:**
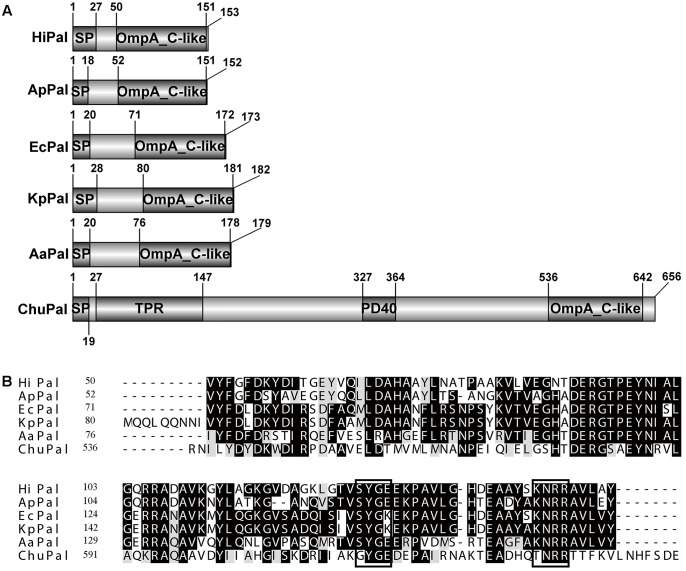
Sequence analysis of CHU_0125 (*chu*Pal). **(A)** Schematic domain architecture of CHU_0125 and related Pals. SP, N-terminal signal peptide. **(B)** Sequence alignment of the OmpA_C-like domain of CHU_0125 and related Pals by CLUSTALW. Dark shading indicated identical amino acids and light shading indicated similar amino acids. Black rectangle box indicated conserved motif in Pals. Their GenBank accession numbers are: *Chu*PAL (*Cytophaga hutchinsonii*) (ABG57418), *Hi*PAL (*Haemophilus influenza*) (NP_438542), *Ap*PAL (*Actinobacillus pleuropneumoniae*) (EFL81759), *Ec*PAL (*Escherichia coli*) (ZP_12055177), *Kp*Pal *Klebsiella pneumoniae* (CDO13556), *Aa*PAL (*Alkalomonas amylolytica*) (AGO28205).

CHU_0125 was annotated as an outer membrane peptidoglycan-associated lipoprotein (Pal) in NCBI. Generally, Pal homologs, such as in *E. coli*, *Haemophilus influenza*, *Actinobacillus pleuropneumoniae, Klebsiella pneumoniae*, and *Alkalomonas amylolytica* only have an OmpA_C-like domain which plays a role in associating with peptidoglycan (**Figure 1A**) (Chen and Henning, 1987; Zlotnick et al., 1988; Frey et al., 1996; Hsieh et al., 2013; Zhai et al., 2014). The full-length of *chu_0125* (GenBank accession number: ABG57418) is 1971 bp and encodes a protein of 656 amino acids. NCBI shows that in addition to the OmpA_C-like domain, CHU_0125 contains two other domains: TPR (tetratricopeptide repeats) and PD40. TPR serves as an interaction module and multiprotein complex mediator (Zeytuni and Zarivach, 2012). PD40 is a WD40-like domain responsible for regulating cellular function such as cell division, cell-fate determination, and mRNA modification (Neer et al., 1994). Sequence alignment revealed that OmpA_C-like domain of CHU_0125 had 40% identity value with Pal from *E. coli* (*Ec*Pal) (**Figure 1B**), but CHU_0125 only had 15% coverage value with *Ec*Pal because of the TPR and PD40 domains (**Figure 1A**).

### Growth Properties of the Δ*0125* Mutant With Glucose as the Carbon Source

**FIGURE 2:**
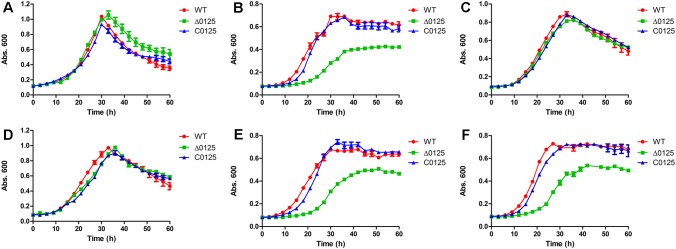
Growth curves of the wild type, the Δ*0125* mutant and the C*0125* in Stainer medium supplemented with 0.4% glucose **(A)**, PY6 medium supplemented with 0.4% glucose **(B)**, PY6 medium supplemented with 0.4% glucose plus 1 g/L KNO_3_ **(C)**, PY6 medium supplemented with 0.4% glucose plus 1 g/L NaNO_3_ **(D)**, PY6 medium supplemented with 0.4% glucose plus 1 g/L KCl **(E)**, PY6 medium supplemented with 0.4% glucose plus 1 g/L NaCl **(F)**. Values are the mean of three biological replicates. Error bars are the standard deviations from these replicates.

To study the function of *chu_0125* in *C. hutchinsonii*, we deleted *chu_0125* and obtained the Δ*0125* mutant (Supplementary Figure S1). Then we complemented the Δ*0125* mutant with pCH0125 and got the C*0125*. The growth properties of the wild type, Δ*0125* mutant, and C*0125* in PY6 and Stainer medium were tested. In Stainer medium, the growth curve of the Δ*0125* mutant was similar to that of the wild type and the C*0125* (**Figure 2A**). However, in PY6 medium, it was found that the Δ*0125* mutant exhibited a long lag period and a reduced biomass at the stationary phase compared with the wild type and the C*0125* (**Figure 2B**). Then we cultured the strains in PY6 medium supplemented with one kind of the inorganic salts of Stainer medium and found that when PY6 medium was supplemented with 1 g/L KNO_3_, the Δ*0125* mutant could grow as well as the wild type (**Figure 2C**), while the other four kinds of inorganic salts of Stainer had almost no effect on the growth of the Δ*0125* mutant (Supplementary Figure S2). To further determine whether K^+^ or NO_3_^-^ played a role in improving the growth of Δ*0125* mutant, KCl and NaNO_3_ were used as the providers of K^+^ and NO_3_^-^, respectively. The growth curves showed that KCl had no effect on the growth of the Δ*0125* mutant (**Figure 2E**) while NaNO_3_ was beneficial to the growth of the Δ*0125* mutant (**Figure 2D**), suggesting that NO_3_^-^ played a role in improving the growth of the Δ*0125* mutant. To eliminate the effect of Na^+^, the growth curves of strains in PY6 medium supplemented with 1 g/L NaCl were monitored, indicating that Na^+^ had no effect on the growth of *C. hutchinsonii* (**Figure 2F**). All these results suggested that the Δ*0125* mutant could not grow as well as the wild type in PY6 medium and additional NO_3_^-^ was important for the growth of the Δ*0125* mutant. In the following tests, we used PY6 medium supplemented with 1 g/L KNO_3_ called PY6K medium to replace the PY6 medium. Compared with PY6 medium, in Stainer and PY6K medium the strains did not have a long stationary phase, which was observed before in our laboratory. However, the reason for this unusual behavior was still unknown.

### Deletion of *chu_0125* Prevents Degradation of the Crystalline Region of Cellulose

**FIGURE 3:**
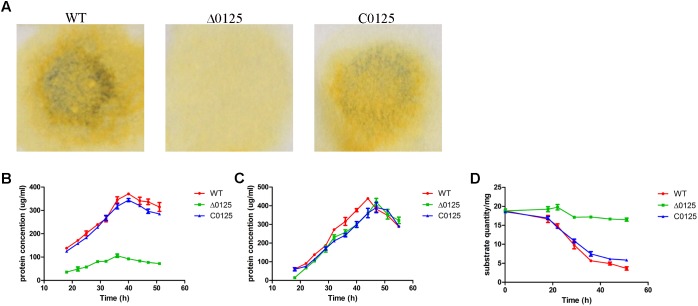
Cellulose degradation assays. Equivalent amounts of middle exponential phase cells were spotted on Whatman NO. 1 filter paper and incubated for 7 days **(A)**. Growth curves of wild type (WT), Δ*0125* mutant (Δ*0125*), and C*0125* in Avicel **(B)** and RAC **(C)** and Avicel utilization rates of wild type, Δ*0125* mutant, and C*0125* **(D)**. Values are the mean of three biological replicates. Error bars are the standard deviations from these replicates.

In order to study the effect of *chu_0125* on cellulose degradation, the ability of *C. hutchinsonii* to digest different kinds of cellulose was monitored. As shown in **Figure 3A**, the wild type and the C*0125* could degrade filter paper while the Δ*0125* mutant was deficient in filter paper degradation. We also measured the growth curves of the wild type, Δ*0125* mutant, and C*0125* when Avicel or RAC was used as the sole carbon source. As shown in **Figure 3B**, the growth rate of the Δ*0125* mutant was lower than that of wild type and C*0125* with Avicel as the sole carbon source while the growth rate of the Δ*0125* mutant was similar to that of wild type and C*0125* with RAC as the sole carbon source (**Figure 3C**). When *C. hutchinsonii* was cultured in Avicel, quantification of the residual Avicel showed that the wild type utilized 81% of the Avicel after incubation for 51 h while the Δ*0125* mutant utilized only 12% of the Avicel in the same time (**Figure 3D**). These results suggested that the Δ*0125* mutant was able to digest RAC as well as the wild type but it was defective in the degradation of cellulose.

**FIGURE 4:**
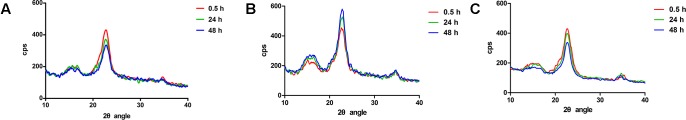
X-ray diffraction spectra of Avicel treated with wild type and Δ*0125* mutant. X-ray diffraction spectra of Avicel treated with wild type **(A)**, Δ*0125* mutant **(B)**, and C*0125* **(C)**. *CI*_XRD_ (%) = (*I*_002_ - *I*_am_)/*I*_002_ × 100%. *I*_002_ is the height of the crystalline peak at 22° and *I*_am_ is the intensity of the peak at 18°.

In addition, the degree of crystallinity of Avicel which was incubated with *C. hutchinsonii* was tested by XRD. After incubation for 48 h, the crystallinity of Avicel was reduced from 65.1 to 57.4% and 58.5% by the wild type and C*0125* respectively (**Figures 4A,C**), while that of the Δ*0125* mutant increased to 70.7% (**Figure 4B**). The result of the increase in crystallinity of Avicel by the Δ*0125* mutant suggested that the Δ*0125* mutant mainly utilized the amorphous region of cellulose when cultured in Avicel. Furthermore, we observed the arrangement of cells on filter paper by scanning electron microscopy (SEM). As shown Supplementary Figure S3A, cells of the wild type grew and arranged on the surface of the fibers, while that of the Δ*0125* mutant grew and gathered in the gully of fibers (Supplementary Figure S3B). Surface morphology of Avicel was also investigated by SEM. The surface of Avicel was smooth and flat after incubated with the wild type (Supplementary Figure S3C) while that of the Δ*0125* mutant was gully shaped (Supplementary Figure S3D). Given our previous finding that the Δ*0125* mutant could mainly degrade the amorphous region of cellulose and was defect in degradation of crystalline region of cellulose, we deduced that the gully shaped surface might be caused by the selective degradation of cellulose by the Δ*0125* mutant.

### Cellulase Activity Determination

**FIGURE 5:**
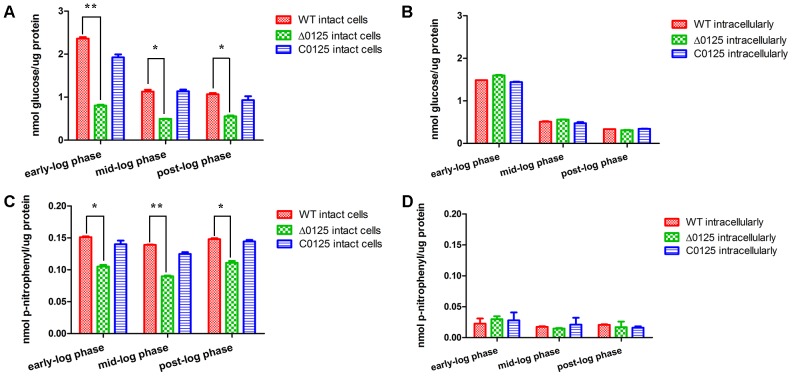
Cellulase activities of wild type (WT), Δ*0125* mutant (Δ*0125*) and C*0125*. Endoglucanase activities of intact cells **(A)** and intracellular **(B)** and β-glucosidase activities of intact cells **(C)** and intracellular **(D)**. All measurements were carried out in triplicate, and error bars indicate standard deviations from these replicates. Significant differences (*T*-test ^∗^*P* < 0.05, ^∗∗^*P* < 0.01) were detected between WT and Δ*0125* mutant.

To study the influence of *chu_0125* on cellulase activity, endoglucanase and β-glucosidase activities of *C. hutchinsonii* were examined. As shown in **Figures 5B,D**, intracellular cellulase activities of the Δ*0125* mutant were almost as same as that of the wild type. But the endoglucanase activity of intact cells of the Δ*0125* mutant was only 40% (the average of three growth phase) of that of the wild type (**Figure 5A**) and β-glucosidase activity of the Δ*0125* mutant was 70% of that of the wild type (**Figure 5C**), indicating that deletion of *chu_0125* affected both endoglucanase and β-glucosidase activities on the cell surface.

### Defect in the Integrity of the Outer Membrane

Studies showed that deletion of *pal* impaired the integrity of outer membrane. To study the function of *chu_0125* in *C. hutchinsonii*, susceptibility of the wild type, Δ*0125* mutant, and C*0125* to some harmful compounds, including sodium dodecyl sulfate, ampicillin, dithiothreitol, H_2_O_2_, and crystal violet, was tested. As shown in **Table 2**, the Δ*0125* mutant was more sensitive to all these reagents than the wild type, suggesting that the outer membrane permeability of the Δ*0125* mutant had been impaired.

**Table 2:** Inhibition zone diameters of wild type and Δ*0125* mutant.

Reagent	Inhibition zone diameter (mm)^a^		
	WT	Δ*0125*	C*0125*
Sodium dodecyl sulfate	24.6 ± 0.9	30.4 ± 0.4^∗^	27.6 ± 0.5
Ampicillin	44.2 ± 0.1	53.7 ± 0.2^∗^	48.3 ± 0.5
Crystal violet	25.0 ± 0.8	30.0 ± 0.8^∗^	27.6 ± 0.9
Dithiothreitol	25.6 ± 0.4	32.0 ± 0.6^∗^	25.3 ± 1.2
H_2_O_2_	37.2 ± 0.8	45.7 ± 0.8^∗∗^	37.7 ± 1.2

^a^*The inhibition zone diameters were the average of three triplicates ± standard deviation. Statistical analysis was carried out using the Student’s *t*-test analysis. Significant differences (*T*-test ^∗^*P* < 0.05, ^∗∗^*P* < 0.01) were detected between WT and Δ*0125* mutant*.

**FIGURE 6:**
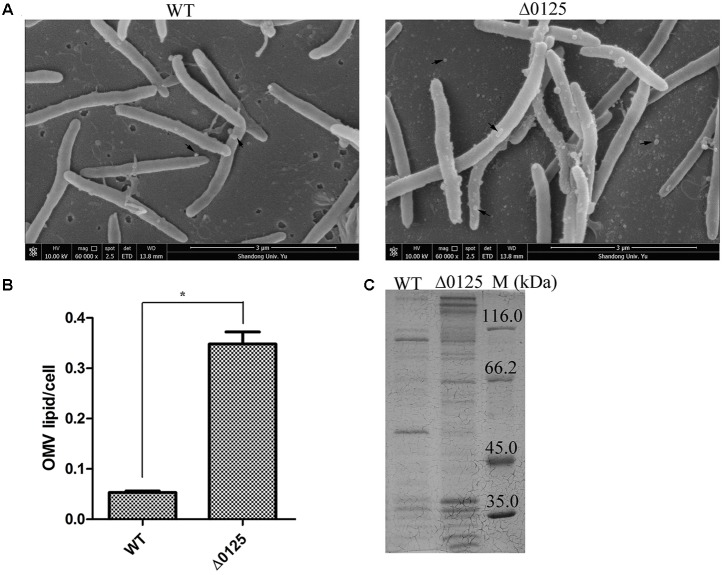
OMVs of wild type (WT) and Δ*0125* mutant (Δ*0125*). Observation of OMVs by SEM **(A)**. Quantification of OMVs **(B)**. SDS-PAGE of OMV proteins **(C)**. The loading samples were normalized by calibration of cell biomass. Quantification of OMVs was carried out in triplicate, and error bars indicate standard deviations from these replicates. Significant differences (*T*-test ^∗^*P* < 0.05, ^∗∗^*P* < 0.01) were detected between WT and Δ*0125* mutant.

The OMVs of the wild type and the Δ*0125* mutant were also observed by SEM. As shown in **Figure 6A**, both the wild type and the Δ*0125* mutant were able to secret OMVs. But the surface of the Δ*0125* mutant was rougher and more OMVs were observed compared with the wild type. Moreover, we quantified the productions of OMVs and found that the OMV production of the wild type was only about 15% of that of the Δ*0125* mutant (**Figure 6B**). We also analyzed the cargo proteins of OMVs by SDS-PAGE. OMVs of the Δ*0125* mutant contained far more kinds of proteins than those of the wild type (**Figure 6C**).

Considering the difference of the outer membrane permeability, OMV productions, and OMV proteins between wild type and Δ*0125* mutant, we deduced that the outer membrane intergity of the Δ*0125* mutant was impaired.

### Analysis of Outer Membrane Proteins

**FIGURE 7:**
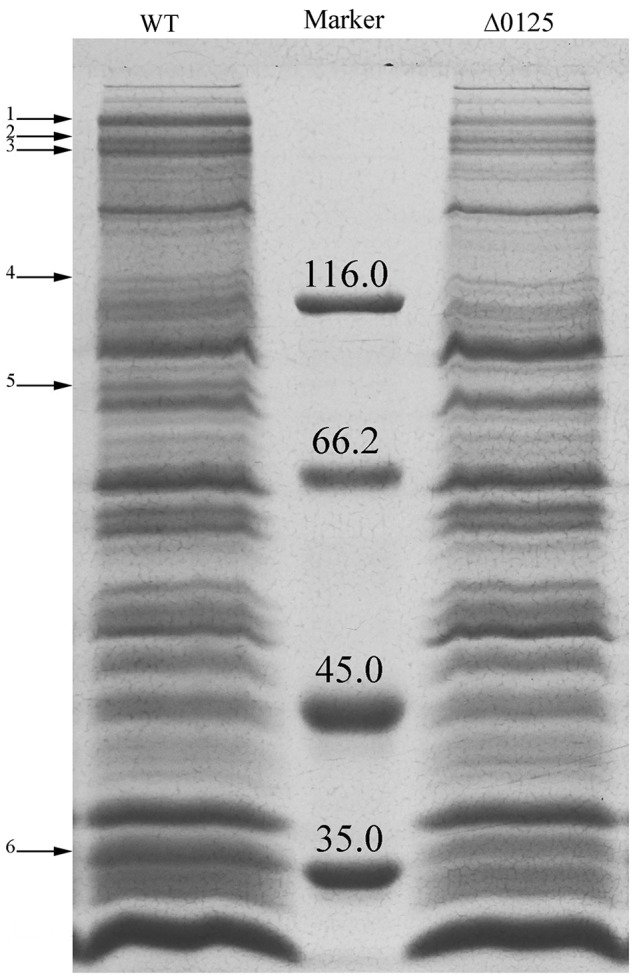
SDS-PAGE of outer membrane proteins of the wild type (WT) and the Δ*0125* mutant (Δ*0125*). All the loading samples were normalized by calibration of cell biomass.

Previous studies showed that defects in outer membrane integrity also resulted in release of outer membrane proteins (Bernadac et al., 1998; Llamas et al., 2000). To determine the changes of the Δ*0125* mutant in outer membrane proteins, we prepared the outer membrane fractions and examined by SDS-PAGE. As shown in **Figure 7**, compared with the wild type, several bands of outer membrane proteins were weakened or missing in the protein profile of the Δ*0125* mutant, which were identified by MALDI-TOF mass spectrometry and listed in **Table 3**. One of the weakened outer membrane proteins was CHU_3220, which was reported to be necessary for the degradation of the crystalline region of cellulose (Wang et al., 2017). Zhu et al. (2013, 2016) reported that disruption of *chu_1107* caused no effect on cellulose degradation. The other three genes, *chu_1075*, *chu_3437*, and *chu_0522*, were singly deleted and it was found that these mutants were able to degrade cellulose as well as the wild type (Supplementary Figure S4). Arrow 5 was identified as CHU_0125, suggesting that CHU_0125 was located on the outer membrane. The disappearance of the band of CHU_0125 in the Δ*0125* mutant also confirmed that *chu_0125* was successfully deleted in the Δ*0125* mutant.

**Table 3:** Identification of differential outer membrane proteins between wild type and Δ*0125* mutant.

Band^a^	CHU no.	Description	MW (kDa)	p*I*
1	CHU_1075	β-glycosidase-like protein	274.2	5.09
2	CHU_3437	Hypothetical protein	245.6	4.82
3	CHU_3220	Hypothetical protein	198.9	4.97
4	CHU_1107	Endoglucanase	135.2	5.39
5	CHU_0125	Outer membrane peptidoglycan-associated lipoprotein	73.8	5.55
6	CHU_0522	Hypothetical protein	29.1	7.09

^a^*Band numbers listed here are corresponding to those denoted in **Figure 7***.

### Deletion of *chu_0125* Affected the Localization of CHU_3220

**FIGURE 8:**
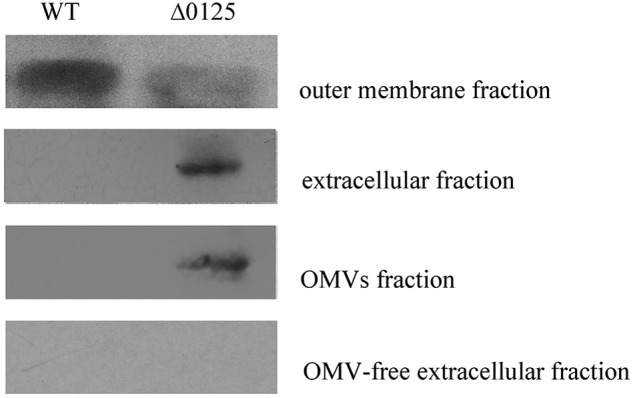
Localization of CHU_3220 in the wild type (WT) and the Δ*0125* mutant (Δ*0125*). Fractions were extracted from wild type and Δ*0125* mutant. All the loading samples were normalized by calibration of cell biomass.

CHU_3220 is a large protein located on the outer membrane and it is the only reported protein to be necessary and specialized for the degradation of the crystalline region of cellulose (Wang et al., 2017). Given the reduced amount of CHU_3220 in the outer membrane of the Δ*0125* mutant as described above, we tested the localization of CHU_3220 in the Δ*0125* mutant. The outer membrane fractions and extracellular fractions were prepared for Western blotting. As shown in **Figure 8**, CHU_3220 could be detected both in the outer membrane fractions of the wild type and the Δ*0125* mutant. However, the amount of CHU_3220 in the outer membrane of the Δ*0125* mutant was about 25% of the amount of wild type as analyzed by Gel-Pro analyzer. In extracellular fractions, the band of CHU_3220 was obvious in the Δ*0125* mutant but it was not observed in the wild type. The results suggested that deletion of *chu_0125* led to CHU_3220 being largely leaked from outer membrane into extracellular milieu. To detect the exact location of CHU_3220 in the extracellular fractions, we prepared OMV fractions and OMV-free extracellular fractions. In the Δ*0125* mutant, CHU_3220 was detectable in OMV fractions and undetectable in OMV-free extracellular fractions which implied that CHU_3220 leaked from outer membrane and was packed into OMVs. Analysis of the OMV proteins by LC-MS/MS found that the unique peptides of CHU_3220 could be detected in the Δ*0125* mutant while they could not be detected in that of the wild type, which was in accordance with the result of Western blotting. All these suggested that in the wild type, CHU_3220 was located on the outer membrane, while in the Δ*0125* mutant a lot of CHU_3220 leaked from outer membrane and was packed into OMVs.

## Discussion

*Cytophaga hutchinsonii* is a widely distributed gliding cellulolytic bacterium, which belongs to the phylum *Bacteroidetes* of Gram-negative bacteria (Stanier, 1942). Proteins of the Pal family are ubiquitous in Gram-negative bacteria, interacting with peptidoglycan and forming the Tol-Pal complex to maintain the integrity of the cell envelop (Lloubes et al., 2001). Mutation of *pal* results in various changes, such as damage of the outer membrane permeability and formation of outer membrane vesicles (Bernadac et al., 1998; Kowata et al., 2016). Sequence alignment showed that the OmpA_C-like domain of CHU_0125 had a 40% identity value with *Ec*Pal. Our results showed that the Δ*0125* mutant was hypersensitive to some harmful compounds. Meanwhile, deletion of *chu_0125* resulted in the mutant producing about 6-fold more OMVs than the wild type (**Figure 6B**). Llamas et al. (2003) reported that the Tol-Pal complex was necessary for appropriate function of certain uptake system. We found that compared with the wild type, the Δ*0125* mutant exhibited a long lag phase and a reduced biomass at the stationary phase in organic medium (PY6 medium) but grew normally in inorganic medium (Stainer medium). When the PY6 medium was supplemented with 1 g/L NO_3_^-^, the growth of Δ*0125* mutant recovered (**Figure 2**). We deduced that limited uptake of some nutrients might be responsible for the hypotrophy of the Δ*0125* mutant in PY6 medium.

We further analyzed the domain structure of CHU_0125, which was found to be different from the domain structures of reported Pals. The reported Pals only possessed an OmpA_C-like domain while CHU_0125 had two other domains, TPR and PD40 (**Figure 1A**). We also analyzed the putative Pals in other bacteria in the phylum *Bacteroidetes*, including *Porphyromonas gingivalis*, *Flavobacterium johnsoniae*, *Flavobacterium columnare*, *Pontibacter indicus*, *Hymenobacter sedentarius*, and *Aquimarina megaterium* and found that they all possessed other domains in addition to the OmpA_C-like domain, suggesting the putative Pals in the phylum *Bacteroidetes* might be different from other reported Pals. Moreover, Cascales et al. reported that the C-terminal SYGK/E motif, a TolA box, was required for the binding of Pal to TolA in *E. coli* (Cascales and Lloubes, 2004). The KNRR motif was also needed for Pal to interact with cell-envelope proteins in *E. coli* (Cascales and Lloubes, 2004). These two motifs were also conserved in some reported Pals (**Figure 1B**). But the two motifs were changed to GYGE and TNRR in CHU_0125 respectively, suggesting that CHU_0125 might interact with other proteins in a different way from *Ec*Pal (**Figure 1B**). In addition to these differences in protein structures, some phenotypes of the Δ*0125* mutant were also different from those of Δ*pal* in *E. coli*. In *E. coli*, deletion of *pal* causes a motility defect (Santos et al., 2014). However, in *C. hutchinsonii*, cells of the Δ*0125* mutant could spread as normally as the wild type on soft agar (Supplementary Figure S5). Gerding et al. (2007) revealed that the Tol-Pal complex was a part of the cell division machinery, but the Δ*0125* mutant did not exhibit cell division deficit as observed by SEM. In addition, we deleted *tolA* (*chu_0135*) and *tolB* (*chu_1429*) and found that the Δ*0135* mutant and the Δ*1429* mutant could digest filter paper and grew in PY6 medium as same as the wild type, indicating that the integrity of the Tol-Pal complex was not necessary for cellulose degradation in *C. hutchinsonii* (Supplementary Figure S6).

Previous study showed that *C. hutchinsonii* appeared to use a contact-dependent digestion strategy, indicating the importance of the outer membrane which directly contacted with cellulose in cellulose degradation. However, only several outer membrane proteins, including CHU_1276, CHU_1277, CHU_0170, and CHU_3220, were reported to be important for the digestion of cellulose. In this study, we found that an outer membrane protein CHU_0125 was necessary and specialized for the degradation of the crystalline region of cellulose by *C. hutchinsonii*. Deletion of *chu_0125* impaired the integrity of outer membrane and this impairment of the outer membrane integrity resulted in the decrease of some outer membrane proteins in the Δ*0125* mutant (**Figure 7** and **Table 3**). Genes of these proteins listed in **Table 3** were singly deleted, but the mutants were able to digest cellulose as same as the wild type except the Δ*3220* mutant. Our previous study showed that the Δ*3220* mutant could not digest crystalline region of cellulose, which was similar to the phenotype of the Δ*0125* mutant in degradation of cellulose (Wang et al., 2017). In addition, Western blotting showed that amounts of CHU_3220 were leaked from outer membrane and packaged into OMVs in the Δ*0125* mutant. These results indicated that deletion of *chu_0125* influenced the localization of CHU_3220, which might affect the degradation of the crystalline region of cellulose by the Δ*0125* mutant. But how the misplaced CHU_3220 affected the cellulose degradation of the Δ*0125* mutant needed to be further studied.

## Conclusion

This work revealed that deletion of *chu_0125* which encoded a putative outer membrane peptidoglycan-associated lipoprotein prevented degradation of the crystalline region of cellulose, which improved our understanding of the function of Pal. This study also enhanced the importance of the integrity of outer membrane in the degradation of the crystalline region of cellulose by *C. hutchinsonii*.

## Author Contributions

XW, ZW, XB, and XL conceived and designed the experiments. XW, ZW, and XB performed the experiments. XW, XB, and WZ analyzed the data. XW, ZW, XB, YZ, WZ, and XL wrote the paper. XW, XB, and XL revised the manuscript. All authors read and approved the final manuscript.

## Conflict of Interest Statement

The authors declare that the research was conducted in the absence of any commercial or financial relationships that could be construed as a potential conflict of interest.

## Footnotes

1 https://www.ncbi.nlm.nih.gov/

2 http://www.genome.jp/tools-bin/clustalw

3 http://tcoffee.vital-it.ch/apps/tcoffee/index.html

4 http://www.ebi.ac.uk/Tools/msa/muscle/

5 http://www.ch.embnet.org/software/BOX_form.html

## References

[B1] Bai X. Wang X. Wang S. Ji X. Guan Z. Zhang W. (2017a). Functional studies of beta-glucosidases of *Cytophaga hutchinsonii* and their effects on cellulose degradation. *Front. Microbiol.* 8:140. 10.3389/fmicb.2017.00140 28210251 PMC5288383

[B2] Bai X. Zhu S. Wang X. Zhang W. Liu C. Lu X. (2017b). Identification of a *fabZ* gene essential for flexirubin synthesis in *Cytophaga hutchinsonii*. *FEMS Microbiol. Lett.* 364:fnx197. 10.1093/femsle/fnx197 28961729

[B3] Bayer E. A. Belaich J. P. Shoham Y. Lamed R. (2004). The cellulosomes: multienzyme machines for degradation of plant cell wall polysaccharides. *Annu. Rev. Microbiol.* 58 521–554. 10.1146/annurev.micro.57.030502.091022 15487947

[B4] Bayer E. A. Lamed R. (1992). The cellulose paradox: pollutant par excellence and/or a reclaimable natural resource? *Biodegradation* 3 171–188. 1369234 10.1007/BF00129082

[B5] Bernadac A. Gavioli M. Lazzaroni J. C. Raina S. Lloubes R. (1998). *Escherichia coli* tol-pal mutants form outer membrane vesicles. *J. Bacteriol.* 180 4872–4878. 9733690 10.1128/jb.180.18.4872-4878.1998 PMC107512

[B6] Bradford M. M. (1976). A rapid and sensitive method for the quantitation of microgram quantities of protein utilizing the principle of protein-dye binding. *Anal. Biochem.* 72 248–254. 10.1016/0003-2697(76)90527-3 942051

[B7] Cascales E. Lloubes R. (2004). Deletion analyses of the peptidoglycan-associated lipoprotein Pal reveals three independent binding sequences including a TolA box. *Mol. Microbiol.* 51 873–885. 10.1046/j.1365-2958.2003.03881.x 14731286

[B8] Chen R. Henning U. (1987). Nucleotide sequence of the gene for the peptidoglycan-associated lipoprotein of *Escherichia coli* K12. *Eur. J. Biochem.* 163 73–77. 10.1111/j.1432-1033.1987.tb10738.x 3545827

[B9] Frey J. Kuhnert P. Villiger L. Nicolet J. (1996). Cloning and characterization of an *Actinobacillus pleuropneumoniae* outer membrane protein belonging to the family of PAL lipoproteins. *Res. Microbiol.* 147 351–361. 10.1016/0923-2508(96)84710-3 8763621

[B10] Gerding M. A. Ogata Y. Pecora N. D. Niki H. de Boer P. A. (2007). The trans-envelope Tol-Pal complex is part of the cell division machinery and required for proper outer-membrane invagination during cell constriction in *E. coli*. *Mol. Microbiol.* 63 1008–1025. 10.1111/j.1365-2958.2006.05571.x 17233825 PMC4428343

[B11] Godlewska R. Wisniewska K. Pietras Z. Jagusztyn-Krynicka E. K. (2009). Peptidoglycan-associated lipoprotein (Pal) of Gram-negative bacteria: function, structure, role in pathogenesis and potential application in immunoprophylaxis. *FEMS Microbiol. Lett.* 298 1–11. 10.1111/j.1574-6968.2009.01659.x 19519769

[B12] Gujrati V. Kim S. Kim S. H. Min J. J. Choy H. E. Kim S. C. (2014). Bioengineered bacterial outer membrane vesicles as cell-specific drug-delivery vehicles for cancer therapy. *ACS Nano* 8 1525–1537. 10.1021/nn405724x 24410085

[B13] Hsieh P. F. Liu J. Y. Pan Y. J. Wu M. C. Lin T. L. Huang Y. T. (2013). *Klebsiella pneumoniae* peptidoglycan-associated lipoprotein and murein lipoprotein contribute to serum resistance, antiphagocytosis, and proinflammatory cytokine stimulation. *J. Infect. Dis.* 208 1580–1589. 10.1093/infdis/jit384 23911714

[B14] Ji X. Bai X. Li Z. Wang S. Guan Z. Lu X. (2013). A novel locus essential for spreading of *Cytophaga hutchinsonii* colonies on agar. *Appl. Microbiol. Biotechnol.* 97 7317–7324. 10.1007/s00253-013-4820-2 23579728

[B15] Ji X. Wang Y. Zhang C. Bai X. Zhang W. Lu X. (2014). Novel outer membrane protein involved in cellulose and cellooligosaccharide degradation by *Cytophaga hutchinsonii*. *Appl. Environ. Microbiol.* 80 4511–4518. 10.1128/AEM.00687-14 24837387 PMC4148786

[B16] Ji X. Xu Y. Zhang C. Chen N. Lu X. (2012). A new locus affects cell motility, cellulose binding, and degradation by *Cytophaga hutchinsonii*. *Appl. Microbiol. Biotechnol.* 96 161–170. 10.1007/s00253-012-4051-y 22543350

[B17] Kowata H. Tochigi S. Kusano T. Kojima S. (2016). Quantitative measurement of the outer membrane permeability in *Escherichia coli* lpp and tol-pal mutants defines the significance of Tol-Pal function for maintaining drug resistance. *J. Antibiot.* 69 863–870. 10.1038/ja.2016.50 27168313

[B18] Lazzaroni J. C. Portalier R. (1992). The excC gene of *Escherichia coli* K-12 required for cell envelope integrity encodes the peptidoglycan-associated lipoprotein (PAL). *Mol. Microbiol.* 6 735–742. 10.1111/j.1365-2958.1992.tb01523.x 1574003

[B19] Llamas M. A. Ramos J. L. Rodriguez-Herva J. J. (2000). Mutations in each of the tol genes of *Pseudomonas putida* reveal that they are critical for maintenance of outer membrane stability. *J. Bacteriol.* 182 4764–4772. 10.1128/JB.182.17.4764-4772.2000 10940016 PMC111352

[B20] Llamas M. A. Rodriguez-Herva J. J. Hancock R. E. Bitter W. Tommassen J. Ramos J. L. (2003). Role of *Pseudomonas putida* tol-oprL gene products in uptake of solutes through the cytoplasmic membrane. *J. Bacteriol.* 185 4707–4716. 10.1128/JB.185.16.4707-4716.2003 12896989 PMC166457

[B21] Lloubes R. Cascales E. Walburger A. Bouveret E. Lazdunski C. Bernadac A. (2001). The Tol-Pal proteins of the *Escherichia coli* cell envelope: an energized system required for outer membrane integrity? *Res. Microbiol.* 152 523–529. 10.1016/S0923-2508(01)01226-8 11501670

[B22] Lynd L. R. Weimer P. J. van Zyl W. H. Pretorius I. S. (2002). Microbial cellulose utilization: fundamentals and biotechnology. *Microbiol. Mol. Biol. Rev.* 66 506–577. 10.1128/MMBR.66.3.506-577.2002 12209002 PMC120791

[B23] Malanovic N. Lohner K. (2016). Gram-positive bacterial cell envelopes: the impact on the activity of antimicrobial peptides. *Biochim. Biophys. Acta* 1858 936–946. 10.1016/j.bbamem.2015.11.004 26577273

[B24] Nandakumar M. P. Cheung A. Marten M. R. (2006). Proteomic analysis of extracellular proteins from *Escherichia coli* W3110. *J. Proteome Res.* 5 1155–1161. 10.1021/pr050401j 16674104

[B25] Neer E. J. Schmidt C. J. Nambudripad R. Smith T. F. (1994). The ancient regulatory-protein family of WD-repeat proteins. *Nature* 371 297–300. 10.1038/371297a0 8090199

[B26] Nur A. Hirota K. Yumoto H. Hirao K. Liu D. Takahashi K. (2013). Effects of extracellular DNA and DNA-binding protein on the development of a *Streptococcus intermedius* biofilm. *J. Appl. Microbiol.* 115 260–270. 10.1111/jam.12202 23551549

[B27] Parsons L. M. Lin F. Orban J. (2006). Peptidoglycan recognition by Pal, an outer membrane lipoprotein. *Biochemistry* 45 2122–2128. 10.1021/bi052227i 16475801

[B28] Santos T. M. Lin T. Y. Rajendran M. Anderson S. M. Weibel D. B. (2014). Polar localization of *Escherichia coli* chemoreceptors requires an intact Tol-Pal complex. *Mol. Microbiol.* 92 985–1004. 10.1111/mmi.12609 24720726 PMC4222177

[B29] Schwechheimer C. Kuehn M. J. (2015). Outer-membrane vesicles from gram-negative bacteria: biogenesis and functions. *Nat. Rev. Microbiol.* 13 605–619. 10.1038/nrmicro3525 26373371 PMC5308417

[B30] Stanier R. Y. (1942). The Cytophaga group: a contribution to the biology of myxobacteria. *Bacteriol. Rev.* 6 143–196. 16350082 10.1128/br.6.3.143-196.1942 PMC440862

[B31] Stewart J. C. M. (1980). Colorimetric determination of phospholipids with ammonium ferrothiocyanate. *Anal. Biochem.* 104 10–14. 10.1016/0003-2697(80)90269-9 6892980

[B32] Wang S. Zhao D. Bai X. Zhang W. Lu X. (2017). Identification and characterization of a large protein essential for degradation of the crystalline region of cellulose by *Cytophaga hutchinsonii*. *Appl. Environ. Microbiol.* 83:e02270-16. 10.1128/AEM.02270-16 27742681 PMC5165126

[B33] Wang Y. Wang Z. Cao J. Guan Z. Lu X. (2014). FLP-FRT-based method to obtain unmarked deletions of CHU_3237 (porU) and large genomic fragments of *Cytophaga hutchinsonii*. *Appl. Environ. Microbiol.* 80 6037–6045. 10.1128/AEM.01785-14 25063660 PMC4178665

[B34] Wessel A. K. Liew J. Kwon T. Marcotte E. M. Whiteley M. (2013). Role of *Pseudomonas aeruginosa* peptidoglycan-associated outer membrane proteins in vesicle formation. *J. Bacteriol.* 195 213–219. 10.1128/JB.01253-12 23123904 PMC3553829

[B35] Wilson D. B. (2009). Evidence for a novel mechanism of microbial cellulose degradation. *Cellulose* 16 723–727. 10.1007/s10570-009-9326-9 18622745

[B36] Xie G. Bruce D. C. Challacombe J. F. Chertkov O. Detter J. C. Gilna P. (2007). Genome sequence of the cellulolytic gliding bacterium *Cytophaga hutchinsonii*. *Appl. Environ. Microbiol.* 73 3536–3546. 10.1128/AEM.00225-07 17400776 PMC1932680

[B37] Xu Y. Ji X. Chen N. Li P. Liu W. Lu X. (2012). Development of replicative oriC plasmids and their versatile use in genetic manipulation of *Cytophaga hutchinsonii*. *Appl. Microbiol. Biotechnol.* 93 697–705. 10.1007/s00253-011-3572-0 21935590

[B38] Zeytuni N. Zarivach R. (2012). Structural and functional discussion of the tetra-trico-peptide repeat, a protein interaction module. *Structure* 20 397–405. 10.1016/j.str.2012.01.006 22404999

[B39] Zhai L. Xue Y. Song Y. Xian M. Yin L. Zhong N. (2014). Overexpression of *Aa*Pal, a peptidoglycan-associated lipoprotein from *Alkalomonas amylolytica*, improves salt and alkaline tolerance of *Escherichia coli* and *Arabidopsis thaliana*. *Biotechnol. Lett.* 36 601–607. 10.1007/s10529-013-1398-9 24249101

[B40] Zhou H. Wang X. Yang T. Zhang W. Chen G. Liu W. (2016). An outer membrane protein involved in the uptake of glucose is essential for *Cytophaga hutchinsonii* cellulose utilization. *Appl. Environ. Microbiol.* 82 1933–1944. 10.1128/AEM.03939-15 26773084 PMC4784033

[B41] Zhu Y. Han L. Hefferon K. L. Silvaggi N. R. Wilson D. B. McBride M. J. (2016). Periplasmic *Cytophaga hutchinsonii* endoglucanases are required for use of crystalline cellulose as sole carbon and energy source. *Appl. Environ. Microbiol.* 82:AEM.01298-16. 10.1128/AEM.01298-16 27260354 PMC4984284

[B42] Zhu Y. Li H. Zhou H. Chen G. Liu W. (2010). Cellulose and cellodextrin utilization by the cellulolytic bacterium *Cytophaga hutchisonii*. *Bioresour. Technol.* 101 6432–6437. 10.1016/j.biortech.2010.03.041 20362433

[B43] Zhu Y. McBride M. J. (2014). Deletion of the *Cytophaga hutchinsonii* type IX secretion system gene sprP results in defects in gliding motility and cellulose utilization. *Appl. Microbiol. Biotechnol.* 98 763–775. 10.1007/s00253-013-5355-2 24257839

[B44] Zhu Y. McBride M. J. (2017). The unusual cellulose utilization system of the aerobic soil bacterium *Cytophaga hutchinsonii*. *Appl. Microbiol. Biotechnol.* 101 7113–7127. 10.1007/s00253-017-8467-2 28849247

[B45] Zhu Y. Zhou H. Bi Y. Zhang W. Chen G. Liu W. (2013). Characterization of a family 5 glycoside hydrolase isolated from the outer membrane of cellulolytic *Cytophaga hutchinsonii*. *Appl. Microbiol. Biotechnol.* 97 3925–3937. 10.1007/s00253-012-4259-x 22790541

[B46] Zlotnick G. W. Sanfilippo V. T. Mattler J. A. Kirkley D. H. Boykins R. A. Seid R. C. Jr. (1988). Purification and characterization of a peptidoglycan-associated lipoprotein from *Haemophilus influenzae*. *J. Biol. Chem.* 263 9790–9794. 3290214

