# Supplementary material for: Deletion of a Gene Encoding a Putative Peptidoglycan-Associated Lipoprotein Prevents Degradation of the Crystalline Region of Cellulose in Cytophaga hutchinsonii
Source: Front Microbiol. 2018 Apr 3;9:632. doi: 10.3389/fmicb.2018.00632 (PMC5891637; doi:10.3389/fmicb.2018.00632)
Supplement: Supplementary file 1 [file Data_Sheet_1.DOCX]

Supplementary Material

**Deletion of a gene encoding a putative** **peptidoglycan-associated lipoprotein prevents** **degradation of the crystalline region of cellulose in *Cytophaga hutchinsonii***

Xifeng Wang^1^, Zhiquan Wang^1^, Xinfeng Bai^2^, Yue Zhao^1^, Weican Zhang^1^, and Xuemei Lu^1^*

1 State Key Laboratory of Microbial Technology, Shandong University, Jinan, China

2 Biology Institute of Shandong Academy of Science/Key Laboratory for Biosensors of Shandong Province, Jinan, China

*Corresponding author. Xuemei Lu

E-mail: [luxuemei@sdu.edu.cn](mailto:luxuemei@sdu.edu.cn)

Tel: +86-531-88369495.

Fax: +86-531-88565610.

**Supplementary Table 1** Sequences of primers used in this study

| Primer | Sequence^a^ |
| --- | --- |
| 0125H1F | ATACGGATCCTTGTAGCAGAACGTGCTTGTTG |
| 0125H1R- | ACTGGGTACCATCACCACCTTCACTTAAAGAC |
| 0125H2F | ATGCGTCGACAGGCAGCGGTTGATTACATTAT |
| 0125H2R | TAGCGAGCTCCCATGAAGTTTACATTTGTCCC |
| 0125UF | AGGATATGGGAAATCAACAC |
| 0125UR | TACGAACAATAACGATACGC |
| 0125DR | ACATCTTTATAGCTCCCGTC |
| 0125CF | ACGCGAGCTCCTGGCTAAATAGATATTTCACG |
| 0125CR | ACGCGTCGACTGCTGCTCATGTTTACTTTATC |
| 0135H1F | ATATGGATCCAAATGGGAGTTTGCTCGGACAG |
| 0135H1R | ATATGGTACCTCGGCGTTATACCAAACCTGTC |
| 0135H2F | ATATGTCGACATTCGGCTAAGAAAGCGGCAAC |
| 0135H2R | ATATGAGCTCGTATTCAGGAAGCAGGTCAAGC |
| 0135UF | GGTTCATTAGGCTCCAGTTC |
| 0135UR | TTTATTTACGACCGGCCGTC |
| 0135DR | CAGCAAGAATAGGGCAAA |
| 1429H1F | ATATGTCGACTATCCATATTGACGTGGGTGGC |
| 1429H1R | ATACCCATGGACCCGAACTACTTCTTCTGGAT |
| 1429H2F | ATAGGGTACCGAACCAAGAGTGATCGCTAAAC |
| 1429H2R | ATAGGGATCCAGTAACATTTGAGCCCTTGGTG |
| 1429UF | AGATTTGGTAGGGCCAGGTA |
| 1429UR | TGTATCCTATGAACCCGAAG |
| 1429DR | ATCAGCCATTGCGAAAGC |
| 0522H1F | ATGGAGCATGCGGTTCCGACCGTTATTG |
| 0522H1R | TTATGAGCTCCCATAAAGCCCGTAAGGGC |
| 0522H2F | CATAGGGTACCATGGGCTCCATGCCATTG |
| 0522H2R | ATGATGGATCCTCACCGCGCAGTATGCCGTG |
| 0522UF | CAAGTGCATTATTGGTGGGATG |
| 0522UR | TTAACAGCAGTTGTATCACACGTTC |
| 0522DR | ACTGATACACGCGAACTGGTATATG |
| 3437H1F | TCAGAGCTCTAGTAGGAAGTGCAACGCCT |
| 3437H1R | TATGTCGACGGTGTTGTTCCAACCTCGTAT |
| 3437H2F | GGGCCATGGATACAAGCTTTACCAGCACA |
| 3437H2R | TGAGGTACCTGTACCATTCCCTAACTGAC |
| 3437UF | ACCAACACCCATTCGCTTAG |
| 3437UR | TCGTAGAACTCGCTGTCG |
| 3437DR | CCGGCTTCAATCTGAATAGCAT |
| 1075H1F | TTCGAGCTCAGTAGGAGGTATTGTTATTAC |
| 1075H1R | AGTGTCGACTTGGCTCAATGCTAACGAAG |
| 1075H2F | CGGGGTACCCAGGATTTATTGCAACTCAAA |
| 1075H2R | CCGGGATCCATGAAAGATACCTTACGC |
| 1075UF | TGTTCAGGCGTGAGTGGT |
| 1075UR | GTTGCGGTTAGATTTACT |
| 1075DR | TACGCTGGATAACGGACA |

^a^ Restriction sites on the primers are underlined.

**Supplementary Figure 1**

**
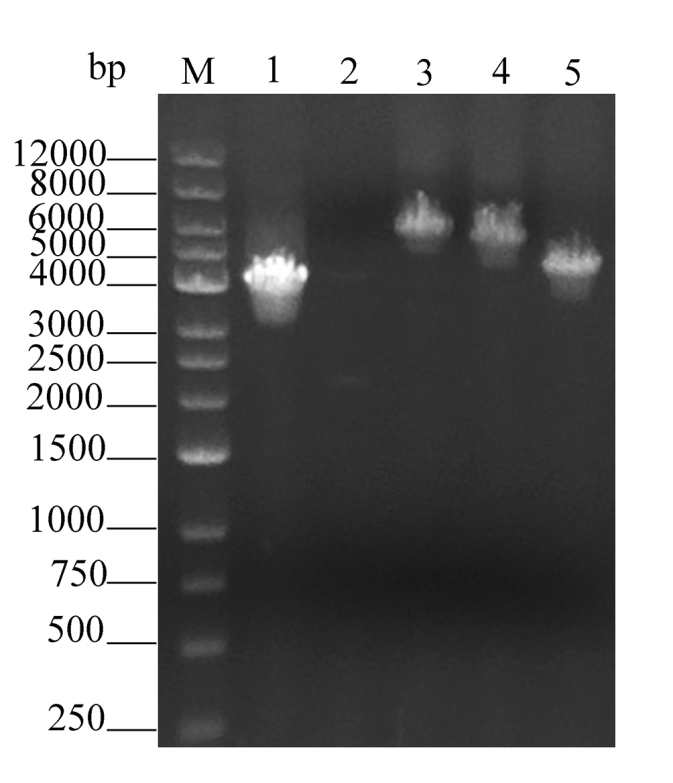
**

**Supplementary Figure 1** PCR verification of the deletion of *chu_0125*. Line 1 and line 3, the wild type; line 2 and line 4, the disruption mutant 0125::*erm*; line 5, the ∆*0125* mutant. Line 1 and line 2 were verified by primers 0125UF/0125UR. Line 3, line 4, and line 5 were verified by primers 0125UF/0125DR.

**Supplementary Figure 2**

**
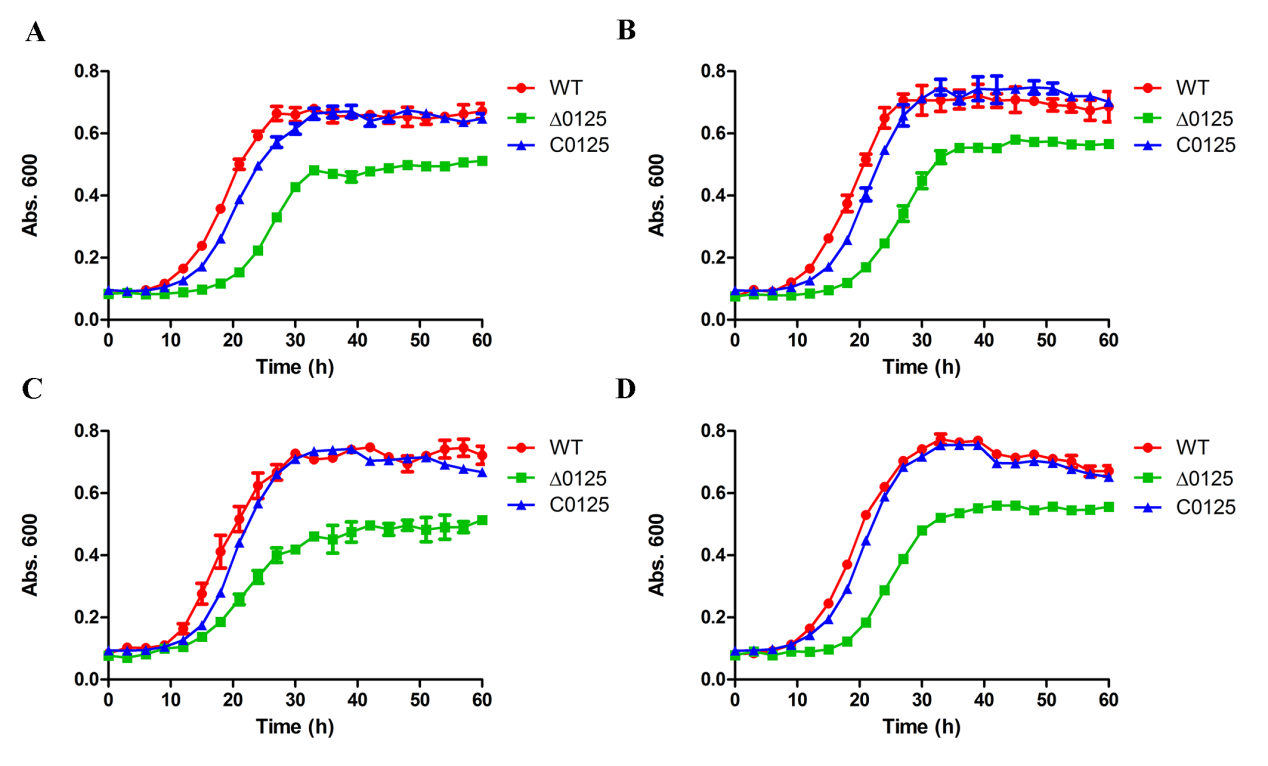
**

**Supplementary Figure 2** Growth curves of the wild type, the ∆*0125* mutant and the C*0125* PY6 medium supplemented with 0.4% glucose plus 1 g/L K_2_HPO_4_ (A), PY6 medium supplemented with 0.4% glucose plus 0.2 g/L MgSO_4_·7H_2_O (B), PY6 medium supplemented with 0.4% glucose plus 0.02 g/L FeCl_3_·6H_2_O (C), PY6 medium supplemented with 0.4% glucose plus 0.1 g/L CaCl_2_ (D). Values are the mean of three biological replicates. Error bars are the standard deviations from these replicates.

**Supplementary Figure 3**


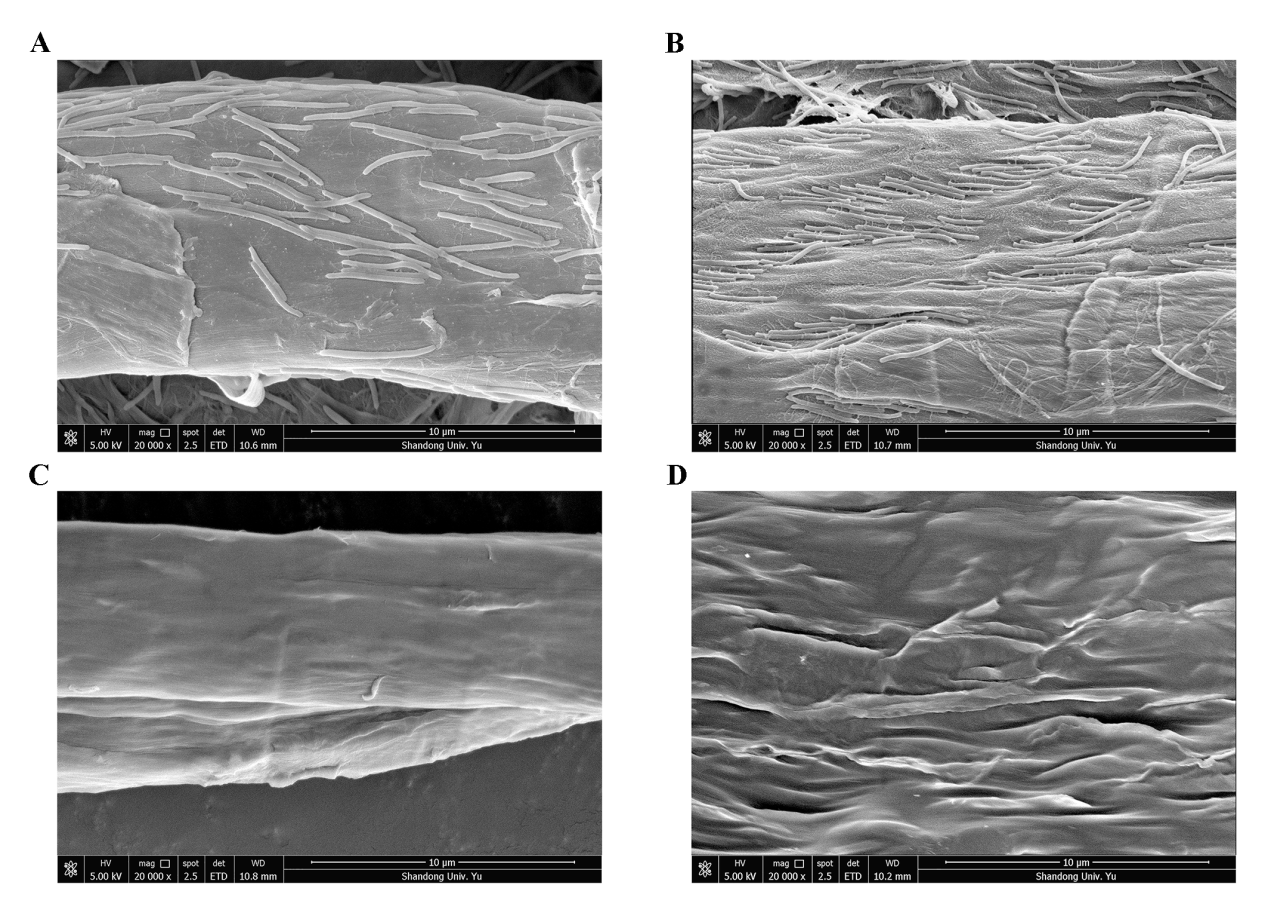


**Supplementary Figure 3** Cells arranged on filter paper and surface morphology of Avicel observed by SEM. Filter paper was digested by wild type (A) and ∆*0125* mutant (B); surface morphology of Avicel after incubated by wild type (C) and ∆*0125* mutant (D).

**Supplementary Figure 4**


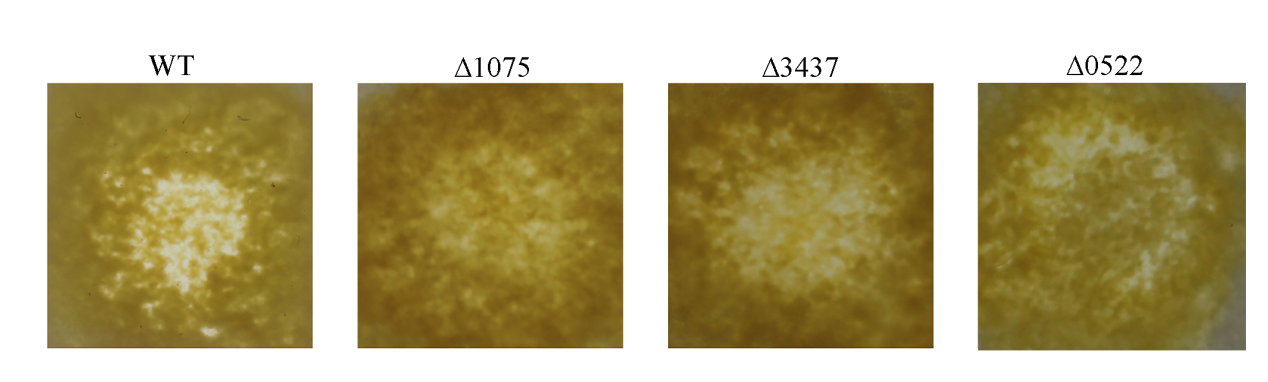


**Supplementary Figure 4** Filter paper degradation assay. Equal amount of cells spotted on Whatman NO. 1 filter paper and incubated at 30 °C. WT, the wild type; ∆*1075*, the ∆*1075* mutant; ∆*3437*, the ∆*3437* mutant; ∆*0522*, the ∆*0522* mutant.

**Supplementary Figure 5**


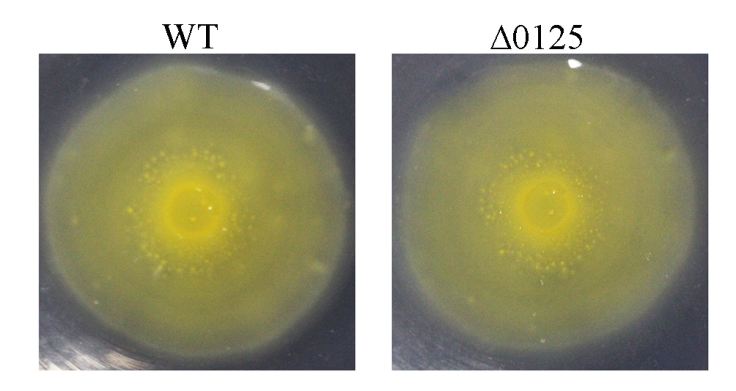


**Supplementary Figure 5** Colony spreading of the cells on soft agar (PY2K medium with 2 g/liter glucose and 5 g/liter agar) at 30°C for 7 days. WT, the wild type; ∆*0125*, the ∆0125 mutant.

**Supplementary Figure 6**


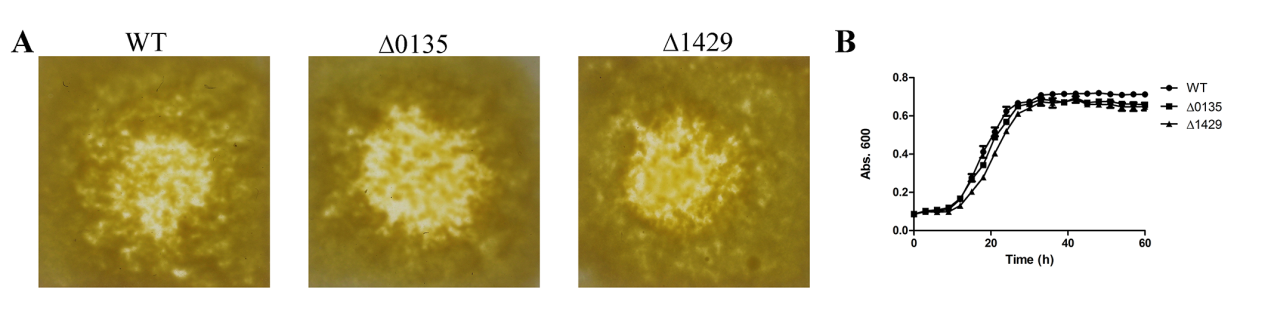


**Supplementary Figure 6** Phenotypes of the ∆*0135* and ∆*1429* mutants. Filter paper degradation assay (A). Equal amount of cells spotted on Whatman NO. 1 filter paper and incubated at 30 °C. Growth curves of these strains in PY6 medium supplemented with 4 g/L glucose (B). WT, the wild type; ∆*0135*, the ∆*0135* mutant; ∆*1429*, the ∆*1429* mutant.
